# From sleeplessness to solitude: emotional repair as a buffer between insomnia and loneliness in university students

**DOI:** 10.3389/frsle.2025.1516094

**Published:** 2025-03-06

**Authors:** Katherine Domar Ostrow, Olivia Rieur, Robert W. Moeller, Martin Seehuus

**Affiliations:** 1 Department of Psychology, Middlebury College, Middlebury, VT, United States,; 2 Department of Psychiatry, Massachusetts General Hospital, Boston, MA, United States,; 3 Department of Oncology, Massachusetts General Hospital, Boston, MA, United States,; 4 Department of Psychological Science, University of Vermont, Vermont Psychological Services, Burlington, VT, United States

**Keywords:** sleep, insomnia, loneliness, emotion regulation, emotional repair, college students

## Abstract

Loneliness and insomnia are endemic in college students, and emotion regulation is strongly related to both. Starting with a biopsychosocial framework, the present study tested a model in which emotional repair mediated the relationship between loneliness and insomnia, with the goal of using a potential mechanism of action to address loneliness. Participants were undergraduate students (N=1,513) in the United States who completed a survey including the Trait Meta-Mood Scale, Sleep Condition Indicator, and UCLA Loneliness Scale, amongst other measures. Insomnia had a significant total negative effect on loneliness, B = −0.46, 95% CI [−0.54, −0.39]. Emotional repair partially mediated this relationship, with an indirect effect of B = 0.015, 95% CI [−0.19, −0.12]. Participants with better sleep were more able to regulate their emotions, and thus tended to experience lower levels of loneliness. Treating insomnia (e.g., CBT–I) or skills associated with emotional repair and regulation (e.g., transdiagnostic approaches to emotion regulation) could reduce overall loneliness.

## 1 Introduction

Over 60% of U.S. college students met the diagnostic criteria for at least one mental health disorder in 2021 (Lipson et al., 2022). College students concurrently report significant levels of insomnia (Gardani et al., 2022; Lund et al., 2010), high levels of loneliness (Demarinis, 2020; Richardson et al., 2017), and many may lack important emotion regulation skills (e.g., Moeller et al., 2020). Research on college students has modeled the relationships between these variables in pairs (e.g., Moeller and Seehuus, 2019; Zou et al., 2020), but little work has explored more complicated connections amongst insomnia, emotion regulation, and loneliness. Given the importance these factors have on overall mental health (Moeller and Seehuus, 2019), understanding the mechanisms by which they interact may help identify potential treatment targets. For example, although research on insomnia and loneliness tends to focus on loneliness as a cause of sleep disruption, this relationship appears to be bidirectional (Hom et al., 2020). Similarly, while insomnia is predictive of emotion regulation (Goldschmied, 2019), emotion regulation has inherent interpersonal and social implications and thus can interfere with effective social interaction, which may impact one’s perception of loneliness (Goldschmied, 2019; Salovey and Mayer, 1990).

The present study frames the relations among insomnia, loneliness, and emotion regulation in college students using the biopsychosocial model. First proposed by Engel (1977), the biopsychosocial model posits that health outcomes are best understood in the context of the overlapping spheres of biological, psychological, and social factors. The three primary variables of interest – sleep (Adams et al., 2020; Becker et al., 2015), loneliness (Luo and Hu, 2022), and emotion regulation (Liu et al., 2023; Perry and Calkins, 2018)—fit well into this bidirectional model and have been thoroughly studied within this context, although the potential mechanisms connecting the three have not been explored.

Insomnia encompasses a multitude of conditions including difficulty falling asleep, challenges staying asleep despite otherwise adequate conditions, or sleep that is experienced as nonrestorative (Roth, 2007). Insomnia is a common challenge for college students (Jiang et al., 2015; Taylor et al., 2013; Williams et al., 2020). The bidirectional relationship between insomnia and physical and psychological health is well-established (Baglioni et al., 2016; Fang et al., 2019; Gradisar et al., 2020; LaVoy et al., 2020; Luyster et al., 2012; Morelhao et al., 2020). Higher levels of insomnia have been associated with decreased subjective well-being (Peach et al., 2016), anxiety and depression (Zou et al., 2020), and increased risk of suicide (Holdaway et al., 2018).

The relationship between sleep and mental health is particularly important to investigate in college students, as this group is at high risk for insomnia due to the dynamic experience of this period of transition. In their meta review, Wang and Bíró (2021) note insomnia was associated with social relationships, stress, caffeine use, lack of regularity in sleep habits, and low levels of physical activity. In addition, college students may not be fully aware of how impaired their sleep is (Peach et al., 2016). For example, in work by Becker et al. (2018) a validated measurement (Pittsburgh Sleep Quality Index, Buysse et al., 1989) classified 62% of their college student sample as poor sleepers, while only 27% of participants self-reported their sleep quality as “fairly bad” or “very bad.”

The feeling of loneliness is a subjective experience of social isolation, or a gap between the companionship a person desires and what they experience (Hawkley and Cacioppo, 2010; Perlman and Peplau, 1981; Russell et al., 1980). This experience of social isolation can be particularly challenging for college students, as the number and proximity of similarly aged peers in the college environment are not necessarily protective factors against loneliness (Moeller and Seehuus, 2019). For people of traditional college age in particular, the experience of loneliness is associated with stigma (Kerr and Stanley, 2021) and shame (Barreto et al., 2022). The stigma associated with loneliness may make it more difficult for college students to admit feelings of loneliness due to self- and/or others’ expectations that one should easily be able to find people to socialize with in college (Bruehlman-Senecal et al., 2020).

While rates of loneliness increased for this group during the COVID-19 pandemic (Labrague et al., 2021), it is difficult to distinguish between a longer-term trend of increasing loneliness and the impact of the pandemic. A meta-analysis from Buecker et al. (2021) found that loneliness has been increasing among emerging adults over the past 43 years (1976–2019). Data from the National College Health Assessment found, of the 24,473 college students who participated in the fall 2023 assessment, ~50% screened positive for loneliness (American College Health Association, 2024).

Loneliness is linked to a variety of clinical psychopathologies in adolescents and emerging adults, including depression (Dunn and Sicouri, 2022) and suicidal ideation (Bennardi et al., 2019; Pereira and Cardoso, 2018). In their prospective study exploring the relationship between loneliness and mental health, Richardson et al. (2017) found higher levels of loneliness at baseline to be predictive of higher rates of depression, stress, and anxiety for university students a year later. Similarly, in a meta-analysis Mann et al. (2022) note that the existing literature points to loneliness as a strong predictor of negative future mental health states, particularly depression, and to a lesser extent anxiety. In light of the existing mental health challenges facing college students, loneliness is an important area of focus.

Cacioppo et al. (2002) found that greater loneliness was linked to higher levels of insomnia among college students. Griffin et al. (2020) sought to elucidate the relation between sleep and loneliness in their meta-analysis; they found that sleep and loneliness were moderately correlated across the 27 studies reviewed, with no meaningful influence of age or gender. While most of the studies included were cross-sectional in nature, the few longitudinal studies included reported mixed findings, leading the authors to conclude that the directionality of the relationship between these two variables is unclear (Griffin et al., 2020).

Insomnia and loneliness have both been linked to depression and anxiety, although the mechanisms by which they are connected are not well studied (Cacioppo and Hawkley, 2003; Cacioppo et al., 2003; Griffin et al., 2020). Loneliness and depression have been shown to predict poor sleep quality and insomnia symptoms (Griffin et al., 2020), but modeling depression and anxiety as predictors of loneliness has not been formally tested, despite evidence suggesting such a relationship (Hom et al., 2017, 2020). More severe insomnia has been linked to heightened feelings of loneliness, but it is unclear the extent to which that relationship is explained by depressive symptoms (Hom et al., 2017).

Sleep also has both direct and indirect effects on social behavior, which may in turn have implications for an individual’s feelings of loneliness (Dorrian et al., 2019). Sleepiness can lead to a decrease in social activity and duration of social activity (Holding et al., 2020), as well as less desire to be in the presence of others and engage in social activities (Axelsson et al., 2020). Similarly, sleep loss has been linked to social avoidance (Simon and Walker, 2018). At the same time, an individual’s sleepiness may deter others from wanting to interact with them. Sleep-deprived participants and photos of such participants, respectively, were rated by independent judges as seeming more lonely (Simon and Walker, 2018; Sundelin et al., 2017). This may be especially detrimental to one’s loneliness levels considering that sleepiness can increase the desire to be cared for by others (Axelsson et al., 2020). Thus, the consequences of insomnia on day-to-day functioning may lead to both increased drive for social contact and impaired ability to obtain or maintain that contact, thus increasing the gap between desired and actual social contact.

Emotional intelligence is the ability to monitor, discriminate between, and skillfully use information about one’s emotions (Salovey and Mayer, 1990). It is also considered a subset of social intelligence (Salovey and Mayer, 1990). Mayer and Salovey (1997) divided emotional intelligence into four related abilities: perceiving, using, understanding, and managing emotions. Emotional intelligence thus encompasses many specific abilities involving one’s own and others’ emotions, including empathy, emotion recognition, emotional expression, emotion identification, and emotion regulation. The Trait Meta-Mood Scale (TMMS; Salovey et al., 1995) is a measure designed to examine various cognitive aspects of emotional intelligence, including emotional repair (Fitness and Curtis, 2005; Salovey et al., 1995). Emotional repair refers to the ability to regulate one’s emotional states and, in particular, to return to baseline from a negative emotional state (Fitness and Curtis, 2005; Salovey et al., 1995).

There is evidence of a bidirectional nature between sleep and one’s ability to regulate, and more specifically repair, their emotional states (Vandekerckhove and Wang, 2018). The repair of one’s emotional states has a strong impact on their ability to settle themself into a state that is conducive to falling and staying asleep (Hoag et al., 2016); therefore, individuals with poor emotional repair are more likely to experience insomnia. Insomnia also lowers the impact of an individual’s ability to repair their emotional states (Baglioni et al., 2010). Thus sleep and emotion regulation, in particular emotional repair, are complexly interconnected, with sleep disruption increasing the need for emotional repair while also impairing the ability to accomplish it.

Similarly, the way in which an individual experiences their emotional states and in particular their ability to modify their emotional experience is an important factor associated with loneliness (Gallardo et al., 2018) and may impact insomnia. In other words, one’s ability to regulate or *repair* their emotions impacts how one interacts with others, which may have implications for experiences of loneliness.

For college students, emotional repair is an important aspect of emotional intelligence and has a strong impact on their mental health (Costa et al., 2013; Jeong et al., 2024), with higher levels of emotional repair associated with significantly lower levels of mental health distress. For example, being able to alter one’s emotions and engage in emotional repair is associated with lower levels of depression (Aradilla-Herrero et al., 2014; Extremera and Fernández-Berrocal, 2006), and emotional repair is also associated with lower levels of anxiety (Extremera and Fernández-Berrocal, 2006; Guil et al., 2021). Other work has suggested that emotional repair may be a mediator between insomnia and depression (Salguero-Alcañiz et al., 2021).

A bidirectional relationship between emotion regulation and loneliness has been demonstrated (Wols et al., 2015; Zysberg, 2012). While some work has focused on specific emotion regulation strategies (Kearns and Creaven, 2017; Preece et al., 2021), other work has explored the connection more broadly, finding that those with chronic loneliness may be less likely to accept invitations for social inclusion and more likely to use maladaptive emotion regulation strategies (Vanhalst et al., 2017). Studies have examined the relationship between emotion regulation and loneliness using the TMMS-repair subscale (Gallardo et al., 2018; Martín-Albo et al., 2015). A study of high school students found experiences of relatedness to be a partial mediator between emotional repair and loneliness, although when relatedness was high, repair directly influenced loneliness (Martín-Albo et al., 2015). Other studies have found that high levels of emotional repair are positively associated with task-oriented coping and negatively associated with destructive and dysfunctional coping (Fitness and Curtis, 2005). High emotional repair has also been found to be associated with positive interactive behaviors and being perceived as more socially competent (Hessel et al., 2016), while low emotional repair may lead to more interpersonal challenges (Trickey et al., 2011). Similarly, Goldschmied (2019) concludes that effectively adapting one’s emotional responses to a given context helps ensure appropriate behavior with others and successful integration into society. In this way, poor emotion regulation may lead to loneliness if it impedes effective interaction with others.

Considering the multiple and complex connections between sleep and emotion regulation (Fairholme and Manber, 2015; Goldschmied, 2019), and the interpersonal implications of impaired emotion regulation (Fitness and Curtis, 2005; Goldschmied, 2019), emotional repair emerges as a possible link between insomnia and loneliness. Among college students, Tavernier and Willoughby (2015) found that sleep disruption contributed to dysfunctional emotion regulation, which in turn resulted in less positive social ties.

The present study uses survey data from a cross-sequential study of mental health among college students. Our aim is to expand the currently limited understanding of the mechanisms linking insomnia to loneliness in a population for which sleep health is particularly important (Becker et al., 2015; Brand and Kirov, 2011) and loneliness is prevalent (Moeller and Seehuus, 2019; U.S. Department of Health Human Services, 2023. We propose that one’s ability to repair negative emotional experiences serves as a mediator between insomnia and loneliness. Thus, we expect our proposed mediation model to find that lower levels of insomnia are associated with better emotional repair (H_1_) and less loneliness (H_2_) and better emotional repair also predicts less loneliness (H_3_). Furthermore, we hypothesize that emotional repair is the mechanism by which insomnia has an effect on loneliness (H_4_).

## 2 Methods

### 2.1 Study design

This article is based on data from a cross-sequential study examining mental health in undergraduate students from two liberal arts colleges in the northeastern United States. Data collection occurred over the span of 2 weeks in January 2022. This study was approved by the Institutional Review Board of the last author’s home institution. An email was sent to all students at both colleges with a unique link to a survey, which was delivered through Qualtrics. Participants indicated their consent online before completing the survey. N = 1,815 completed the survey, for a response rate of 39.77%. Students who completed the survey were entered into a raffle to win one of many Amazon gift cards.

### 2.2 Sample size and power

A power analysis was run to determine if the available sample would likely be able to meaningfully address the research questions. The power analysis focused on the mediation model in which emotional repair mediates the relationship between insomnia and loneliness. We used the R package semPower (Moshagen and Bader, 2023) version 2.1.0 with an alpha value of 0.05 and a beta value of 0.10. Our a priori estimate for the a path from insomnia to emotional repair was set at a conservatively small 0.15, based on the relationships observed using different instruments to measure the same relationship (Emert et al., 2017; Killgore et al., 2022). Our value for the b path from emotional repair to loneliness was set at 0.25, based on existing research addressing a similar mediation model using different measurement instruments (Martín-Albo et al., 2015). Finally, the c’ path from sleep to loneliness was estimated at 0.32 based on a meta-analysis addressing that specific question (Martín-Albo et al., 2015), using the smaller end of the confidence interval for observed relationships. Note that the c’ estimate assumes no mediation, which is a more conservative approach. Given these parameters, we estimate the required number of participants to find effects of approximately the same size as those that previous studies have observed to be 463. The actual raw sample size of *N* = 1,815 exceeds that estimate.

### 2.3 Participants

Data quality checks were conducted prior to analysis. Participants were removed if they did not complete all of the measures used in this survey (*n* = 230), or if they completed the survey in less than the average time minus three standard deviations, or if they had more than one straight-line response pattern (an additional *n* = 51). Thus, the final analytic data set of *N* = 1,534 did not contain any relevant missing data and had passed all data quality checks. No data transformations were conducted prior to analysis. Analyses were run to determine if there were significant differences in demographic or other variables of interest between completers and non-completers, with no significant differences observed. See Table 1 for sample demographics by gender.

### 2.4 Measures

#### 2.4.1 Insomnia

The Sleep Condition Indicator (SCI; Espie et al., 2014) was used to measure insomnia. The SCI is a widely-used, eight-item measure designed to detect signs of insomnia disorder, and has been validated in several languages (Palagini et al., 2015; Uygur et al., 2024). The measure captures sleep onset, maintenance, awakenings, quality, as well as daytime functioning and persistence of sleep problems (Espie et al., 2014). Items include, “Thinking about a typical night in the last month, how long does it take you to fall asleep?”; “Thinking about the past month, to what extent has poor sleep affective your mood, energy, or relationships?”; and “How long have you had a problem with your sleep?” Each item has five options: for instance, the options for the first question listed above are 0–15 min, 16–30 min, 31–45 min, 46–60 min, and ≥61 min, and the options for the second question are “Not at all,” “A little,” “Somewhat,” “Much,” and “Very much.” Responses are then summed with a possible range of 0–32 with a higher score representing better sleep quality. In the present sample, the SCI had a Cronbach’s alpha of 0.86, demonstrating adequate internal reliability.

#### 2.4.2 Emotional repair

Emotional repair was measured using the emotion repair subscale of the Trait Meta-Mood Scale (TMMS) (Salovey et al., 1995). This subscale has 12 items including “If I find myself getting mad, I try to calm myself down” and “When I am upset I realize that the ‘good things in life’ are illusions,” which are measured on a five-point scale with 1 being “strongly disagree” and 5 being “strongly agree.” The repair subscale had adequate reliability in the present sample, with a Cronbach’s alpha of 0.82.

#### 2.4.3 Loneliness

Loneliness was measured with the UCLA Loneliness Scale (version 3) (Russell, 1996). This twenty-item measure is scored on a scale of 1 (“never”) to 4 (“always”), including items such as, “How often do you feel that you lack companionship?” and “How often do you feel that your relationships with others are not meaningful?” This measure demonstrated adequate internal reliability, with an observed Cronbach’s alpha of 0.94.

## 3 Results

Normality of the three relevant variables (insomnia, emotional repair, and loneliness) was assessed by visual analysis of the Q-Q residual plots. No significant deviation from the expected pattern was observed. Correlations between the variables of interest were estimated, with significant correlations observed between loneliness and insomnia (r = −0.30, *p* < 0.001) and emotional repair (r = −0.46, *p* < 0.001), and between insomnia and emotional repair (r = 0.24, *p* < 0.001).

**FIGURE 1:**
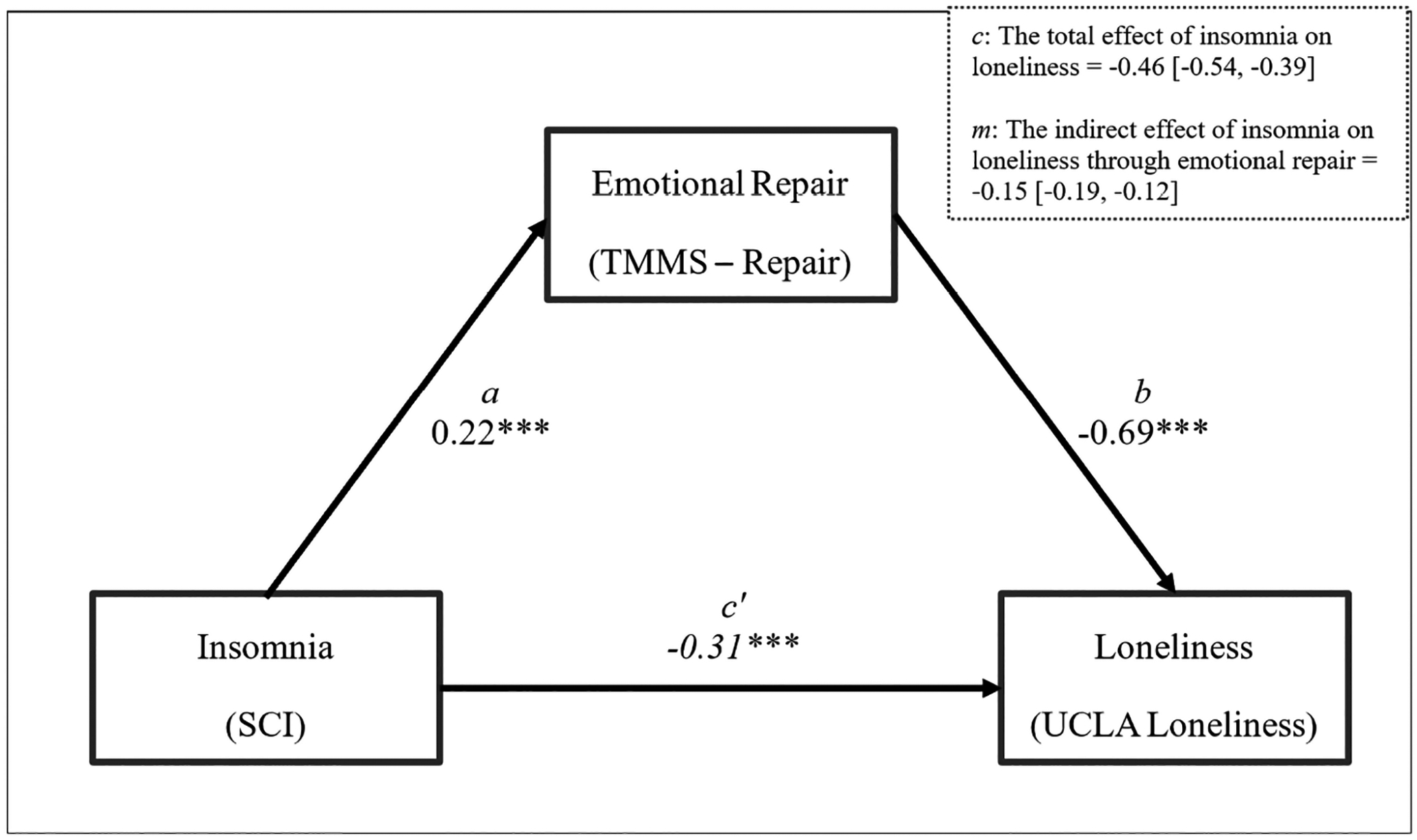
Emotional Repair partially mediates the relationship between sleep quality and loneliness. R^2^ = 0.25; *F*_(2, 1,531)_ = 256.09, *p* < 0.001. *p < 0.05; **p < 0.01; ***p < 0.001. All coefficients are unstandardized. *a* is the effect of insomnia on emotional repair; *b* is the effect of emotional repair on loneliness; *c’* is the direct effect of insomnia on loneliness, while considering the indirect effect through emotional repair.

To test our hypothesis, a mediation analysis was performed in SPSS 29.0 using the PROCESS macro (Hayes, 2022), using Model 4. Regression assumptions were tested, including (a) autocorrelation, assessed via the Durbin-Watson statistic (DW = 1.99), which indicated no meaningful autocorrelation; (b) normality and homoscedasticity of residuals, assessed through both estimation of standardized residuals (M = 0.00; range = −2.83 to 3.63), which fell within acceptable limits, and visual inspection of residual plots confirming no strong deviations from normality or non-constant variance; and (c) multicollinearity, assessed via variance inflation factors (VIF < 1.10 for all predictors), indicating no concerns regarding multicollinearity. These results confirm that the data meet the necessary assumptions for regression analysis. Our results are illustrated in Figure 1 and detailed in Table 2. These results partially support our hypotheses, with the variables of interest (insomnia and emotional repair) accounting for a statistically significant (*p* < 0.001) and meaningful (R^2^ = 0.25) amount of variability in loneliness. As we hypothesized, higher scores on the SCI (representing lower levels of insomnia) are associated with better emotional repair (H_1_, R^2^ = 0.06, *p* < 0.001) as well as with lower levels of loneliness (H_2_, R^2^ = 0.09, *p* < 0.001), and better emotional repair is associated with less reported loneliness (H_3_, R^2^ =0.21, *p* < 0.001). Our mediation hypothesis (H_4_) was partially supported. The total effect of insomnia on loneliness (*c*) was B = −0.46, 95% CI [−0.54, −0.39], but both the direct (*c’*, B = −0.31, 95% CI [−0.38, −0.24]) and indirect pathway (*m*, B = −0.15, 95% CI [−0.19, −0.12]) between insomnia and loneliness account for a meaningful portion of the variance in loneliness. That is, while emotional repair accounts for a significant and meaningful proportion of the overall effect of insomnia on loneliness, other unaccounted for factors remain.

## 4 Discussion

Our results partially support our hypothesized model: We observed a significant, though small, negative association between SCI scores and loneliness, indicating that lower levels of insomnia are related to decreased loneliness. In addition, emotional repair partially mediated the relationship between insomnia and loneliness. The former finding aligns with previous research that has examined the impact of insomnia on loneliness (Simon and Walker, 2018; Hom et al., 2017, 2020), and additionally provide more information about how insomnia and loneliness may be related to each other: through emotional repair. Sleep is related to our emotions in several ways, impacting our affect or mood (Baum et al., 2014; McMakin et al., 2016), empathy (Tempesta et al., 2018), emotion recognition (de Almondes et al., 2016; Tempesta et al., 2018), emotional expression (McGlinchey et al., 2011), and more. The present study brings more attention to one’s ability to manage their emotions and repair a negative emotional state in the context of sleep and social interaction/isolation. Gross’s (2014, 2015) models of emotion regulation suggest that emotion regulation is a complex process requiring significant cognitive resources, and sleep is an essential aspect of strong cognitive functioning; thus, we would expect better sleep quality to be related to heightened emotion regulation ability. At the same time, one’s emotional stability can affect the quality of their social interactions (Fitness and Curtis, 2005; Goldschmied, 2019).

The results of this study provide additional insight into contributing factors associated with college students’ experiences of loneliness. Improving emotional repair skills may be a useful target of intervention for colleges seeking to reduce students’ experiences of insomnia and loneliness. It may be apt, then, to incorporate more opportunities on college campuses for students to learn how to improve their emotional repair skills as a way to buffer against loneliness.

Ellard et al. (2023) conducted a review of 22 articles addressing loneliness interventions in college students and identified four different intervention strategies: psychoeducation, increased social support, reflective exercises, and increased social interaction, the latter three exhibiting greater effectiveness than the former. Considering the interpersonal nature of emotion regulation, a social-based intervention may work particularly well in this context. As the present study suggests, however, targeting students’ sleep may also be a meaningful point of intervention. There have been several examples of effective interventions among college students to improve sleep, most prominently those that are based on cognitive-behavioral therapy (Friedrich and Schlarb, 2018).

### 4.1 Limitations

It is important to note that our model relies on a self-report as opposed to physiological measures of sleep disruption. It is possible that our participants’ self-perceptions of their sleep (as measured by the SCI) are not in agreement with what polysomnography, actigraphy, or other more objective methods would report. Furthermore, mediation implies a temporal order, which our methodology is unable to confirm due to the cross-sectional nature of our data. Even though this model is consistent with our hypotheses, we did not measure any of the variables across time, and therefore these data do not speak directly to temporal directionality. The online nature of our survey could be considered an additional limitation; however evidence suggests that findings from surveys conducted over the internet are consistent with findings from traditional survey methods (e.g., Gosling et al., 2004; Uygur et al., 2022).

### 4.2 Future directions/implications

Considering the limitations of self-report sleep measures and the impracticality of polysomnography for studies of this nature, future research should consider using wearable sleep monitoring devices to track sleep directly. This multi-method approach would allow future research to speak more clearly to the mechanisms of the connections. Furthermore, our findings highlight the potential for loneliness (or more broadly, mental health) interventions that are low-cost and less stigmatizing: that is, by targeting sleep at the individual level. Sleep quality may have advantages over other mental health issues in terms of treatment because sleep dysfunction is relatively low in stigma, and it is a convenient target of intervention. College students may be more willing to participate in such a sleep intervention, due to minimal feelings of shame and/or embarrassment, providing another avenue for intervention. The present study partially explains the relationship between sleep and loneliness, suggesting that emotional repair accounts for some of that connection. These findings may also suggest that, by making more of an effort to prioritize sleep in both self-care and clinical treatment, emotional repair and loneliness may both be improved.

## Figures and Tables

**TABLE 1:** Sample demographics.

Variable	Men	Women	TGD	All
	*N* (%) or M ± SD	*N* (%) or M ± SD	*N* (%) or M ± SD	*N* (%) or M ± SD
Race	χ^2^ (10) = 11.66, *p* = 0.31, V = 0.062			
Native American	0 (0.00%)	4 (0.44%)	0 (0.00%)	4(0.26%)
Asian	51 (9.50%)	122 (13.56%)	10 (11.11%)	183(11.98%)
Black	33 (6.15%)	41 (4.56%)	6 (6.67%)	80(5.24%)
Hawaiian or Pacific Islander	1 (0.19%)	0 (0.00%)	0 (0.00%)	1(0.07%)
White	383 (71.32%)	621 (69.00%)	62 (68.89%)	1,066(69.81%)
Other	69 (12.85%)	112 (12.44%)	12 (13.33%)	193(12.64%)
Ethnicity	χ^2^ (2) = 3.91, *p* = 0.14			
Hispanic	48 (8.89%)	95 (10.51%)	14 (15.56%)	157(10.23%)
Not Hispanic	492 (91.11%)	809 (89.49%)	76 (84.44%)	1,377(89.77%)
Sexual orientation	χ^2^ (6) = 262.69, *p* < 0.001, V = 0.293			
Heterosexual/straight	447 (82.78%)	553 (61.24%)	7 (7.78%)	1,007(65.69%)
Bisexual	32 (5.93%)	191 (21.15%)	28 (31.11%)	251(16.37%)
Gay or lesbian	40 (7.41%)	40 (4.43%)	20 (22.22%)	100(6.52%)
Not listed	21 (3.89%)	119 (13.18%)	35 (38.89%)	175(11.42%)
Year in college	χ^2^ (6) = 6.40, *p* = 0.38			
First year	162 (30.00%)	233 (25.77%)	19 (21.11%)	414(26.99%)
Second year/sophomore	136 (25.19%)	228 (25.22%)	26 (28.89%)	390(25.42%)
Third year/junior	94 (17.41%)	181 (20.02%)	15 (16.67%)	290(18.90%)
Fourth year/senior	148 (27.41%)	262 (28.98%)	30 (33.33%)	440(28.68%)
Perceived socioeconomic status	χ^2^ (4) = 1.72, *p* = 0.79			
Lower	78 (14.44%)	121 (13.38%)	16 (17.78%)	215(14.02%)
Middle	255 (47.22%)	444 (49.12%)	42 (46.67%)	741(48.31%)
Upper	207 (38.33%)	339 (37.50%)	32 (35.56%)	578(37.68%)
Age	20.15 ± 1.43	20.05 ± 1.38	20.14 ± 1.44	20.09 ± 1.41
SCI	22.27_a_ ± 6.60	20.47_b_ ± 7.17	18.84_c_ ± 8.38	21.01 ± 7.12
TMMS repair	29.39_a_ ± 6.08	28.59_b_ ± 6.91	25.81_c_ ± 7.51	28.71 ± 6.71
UCLA loneliness	43.79_a_ ± 11.12	43.66_a_ ± 10.79	49.11_b_ ± 11.42	44.03 ± 11.12

For continuous variables, subscripts indicate differences significant at p < 0.05.

**TABLE 2:** Emotional repair partially mediates the relationship between sleep quality and loneliness.

		Consequent						
		Emotional repair (mediator)				Loneliness (outcome)		
Antecedent		*B*	*SE (B)*	*p*		*B*	*SE (B)*	*p*
Sleep quality (predictor)	*a*	0.22	0.02	<0.001	*c*’	−0.31	0.04	<0.001
					*b*	−0.69	0.04	<0.001
		R^2^ = 0.06			R^2^ = 0.25			
		*F*_(1, 1532)_ = 92.13, *p* < 0.001			*F*_(2, 1531)_ = 256.09, *p* <0.001			
						*B*	95% CI	
Total effect (*c*) of SCI on loneliness						−0.46	[−0.54, −0.39]	
Direct effect (*c*’) of SCI on loneliness						−0.31	[−0.38, −0.24]	
Indirect effect (*m*) of SCI on loneliness through emotional repair						−0.15	[−0.19, −0.12]	

All coefficients are unstandardized.

## Data Availability

The raw data supporting the conclusions of this article will be made available by the authors, without undue reservation.
